# Tracing the Dispersal Pathway of HIV-1 Subtype C to Bahia: Phylogenetic Connections to Southern Brazil

**DOI:** 10.3390/v16121941

**Published:** 2024-12-19

**Authors:** Daniel Polita, Laise de Moraes, Marta Giovanetti, Filipe Ferreira de Almeida Rego, Luciane Amorim Santos, Dennis Maletich Junqueira, Ricardo Khouri

**Affiliations:** 1 Programa de Pós-Graduação em Ciências Biológicas: Bioquímica Toxicológica (PPGBTox), Laboratório de Bioinformática e Evolução Viral, Universidade Federal de Santa Maria (UFSM), Santa Maria 97105-900, Rio Grande do Sul, Brazil; daniel.polita@acad.ufsm.br (D.P.); dennis.maletich@ufsm.br (D.M.J.); 2 Instituto Gonçalo Moniz, Fundação Oswaldo Cruz, Rua Waldemar Falcão, 121, Candeal, Salvador 40296-710, Bahia, Brazil; laise.moraes@fiocruz.br (L.d.M.); luciane.tika@gmail.com (L.A.S.); 3 Programa de Pós-Graduação em Ciências da Saúde, Faculdade de Medicina da Bahia, Universidade Federal da Bahia, Praça Ramos de Queirós, s/n, Largo do Terreiro de Jesus, Salvador 40026-010, Bahia, Brazil; 4 Sciences and Technologies for Sustainable Development and One Health, University of Campus Bio-Medico, 00128 Rome, Italy; giovanetti.marta@gmail.com; 5 René Rachou Institute, Oswaldo Cruz Foundation, Belo Horizonte, Brazil University, Belo Horizonte 31270-901, Minas Gerais, Brazil; 6 Escola Bahiana de Medicina e Saúde Pública, Avenida Dom João VI, 275, Brotas, Salvador 40290-000, Bahia, Brazil; filipefar@hotmail.com; 7 Faculda de Medicina, Universidade Federal da Bahia, Av. Reitor Miguel Calmon, S/N—Vale do Canela, Salvador 40110-100, Bahia, Brazil; 8 Rega Institute for Medical Research, Department of Immunology, Microbiology and Transplantation, KU Leuven, Herestraat 49-Box 1030, 3000 Leuven, Belgium

**Keywords:** HIV-1, Brazil, Subtype C, transmission of infectious disease, molecular phylogenetics, epidemics, Bahia

## Abstract

The HIV-1 epidemic in Brazil is predominantly characterized by subtype B, except in the southern states, where subtype C (HIV-1C) is more prevalent. Continuous monitoring of this profile is essential to maintain an accurate understanding of the molecular landscape of the HIV epidemic in Brazil. In this study, we isolated and sequenced seven new HIV-1C strains from the state of Bahia, located in the Northeast region of Brazil. To reconstruct the phylogenetic history of HIV-1C in the Northeast and investigate its connections with other regions of the country and globally, we first compiled a dataset of 3631 HIV-1C sequences from Brazil, Africa, and Europe. As expected, most of the new HIV-1C sequences from Bahia (n = 6) clustered within the well-known Brazilian clade. However, one sequence from Bahia clustered within the African clade, suggesting a possible new introduction of HIV-1C into Brazil. Furthermore, our findings indicate that the HIV-1C cases in Bahia likely originated from southern states, particularly Santa Catarina. This study provides valuable insights into the molecular profile of the HIV epidemic in Brazil, expanding our understanding of HIV-1C beyond the Southern region.

## 1. Introduction

The HIV epidemic in Brazil exhibits a complex landscape, with significant regional differences in prevalence and subtype distribution. From 2007 to 2023, nearly half a million cases of HIV-1 have been recorded in Brazil [1]. The highest concentration of cases occurred in the Southeast region, accounting for 41.5% of notifications, followed by the Northeast (21.3%), South (19.1%), North (10.2%), and Central-West (7.9%) regions [1]. The genetic diversity of HIV-1 in Brazil is notable, with subtypes B, C, F1, and circulating recombinant forms BC and BF present [2]. Subtype B is the most prevalent variant across the country, except in the Southern region, where subtype C (HIV-1C) predominates [3]. The Northeast region, which accounts for over a fifth of HIV cases in Brazil, presents an epidemic characterized by the predominance of subtype B, (76%) of cases, followed by subtype F1 (8%), subtype C (2%), and recombinant forms (14%) [4]. In addition, recent findings have identified four cases of HIV-1 subtype D in the Northeast region, marking a rare but noteworthy occurrence that may signal new trends in viral subtype diversification within Brazil [5]. Although HIV-1C has historically been uncommon in this region [6], recent studies indicate a potential expansion of this subtype [7,8,9], similar to the pattern observed in the Southeast region of the country [10]. This expansion is accompanied by a decline in subtype B frequency [2], indicating shifts in the dynamics of HIV-1 transmission in Brazil. This study aims to reconstruct the phylogenetic history of HIV-1C circulating in the northeastern state of Bahia, correlating it with the dispersal of this subtype across Brazil.

## 2. Material and Methods

Seven individuals diagnosed with HIV-1C between 2015 and 2021 were enrolled in this study, all receiving care at the Specialized Center for Diagnosis, Assistance, and Research (CEDAP) in Salvador, Bahia, Northeast Brazil. The study adhered to the Declaration of Helsinki and was approved by the Institutional Review Board of the Gonçalo Moniz Institute (IGM-FIOCRUZ) (protocol number 1.764.505).

Viral RNA isolation was performed using a QIAamp Viral RNA Mini kit (Qiagen, Hilden, Germany) following the manufacturer’s instructions. The protease/reverse transcriptase (PR/RT) region was amplified and sequenced according to previously established methods [11]. The polymerase chain reaction (PCR) was conducted using a SuperScript III One-Step RT-PCR system with Platinum Taq DNA Polymerase (Thermo Fisher Scientific, Waltham, MA, USA) and primers K1 (CAGAGCCAACAGCCCCACC) and K2 (TTTCCCCCACTAACTTCTGTATGTCATTGACA) [12]. Internal PCR employed Platinum Taq DNA Polymerase High Fidelity (Thermo Fisher Scientific, USA) with primers DP16 (CCTCAATCACTCTTTGGCAAC) and RT4 (AGTTCATAACCCATCCAAAG) [13]. Internal PCR products were subsequently sequenced using an ABI 3500xL Genetic Analyzer (Applied Biosystems, Waltham, MA, USA). Sequence visualization, editing, and assembly were completed using Geneious v.10.0.8 software. Subtyping was determined using the REGA HIV-1 Subtyping Tool v.3.46 available on Genome Detective (https://www.genomedetective.com). Final sequences encompassed 1003 base pairs (bp) from the pol gene region (2253–3254 relative to HXB2).

We proceeded to compile an initial dataset of 12,558 HIV-1C sequences, comprising 7 sequences obtained in the present study: 3285 sequences were identified through a BLAST search using these sequences as query, and 9266 subtype C sequences from Brazil were retrieved from the Los Alamos National Laboratory database. After applying exclusion criteria to eliminate sequences with ambiguities, duplicates, non-subtype C, evidence of recombination, excessive shortness, poor alignment, and incomplete metadata a total of 3631 sequences were selected for phylogenetic analysis. Phylogenetic reconstruction was performed using the maximum likelihood (ML) method implemented in IQ-TREE v1.6.12 [14]. The best-fit substitution model (GTR + F + I + G4) was selected using ModelFinder [15] prior to phylogenetic reconstruction. Branch support was calculated using the approximate likelihood-ratio (aLRT) SH-like test. The resulting ML tree was visualized, manipulated, and edited using FigTree v1.4.4. To identify the geographic origins and the temporal dynamics of HIV-1C in the state of Bahia, the phylogenetic tree was analyzed in TreeTime v.1.1.1 [16] under a ‘mugration’ model.

## 3. Results and Discussion

Demographic and clinical data of the patients enrolled in this study are summarized in Table 1. The cohort consisted of three females and four males, with ages ranging from 26 to 44 years. The CD4 cell counts varied significantly, from 43 cells/mm^3^ in a 44-year-old female (HV0461) to 455 cells/mm^3^ in a 27-year-old male (HV0312). Viral load also showed marked variation, with the highest recorded at 256,632 copies/mL (HV0461) and the lowest at 407 copies/mL (HV0323), both observed in female individuals. Data on viral load and CD4 counts were not available for two male individuals due to the lack of follow-up.

**Figure 1:**
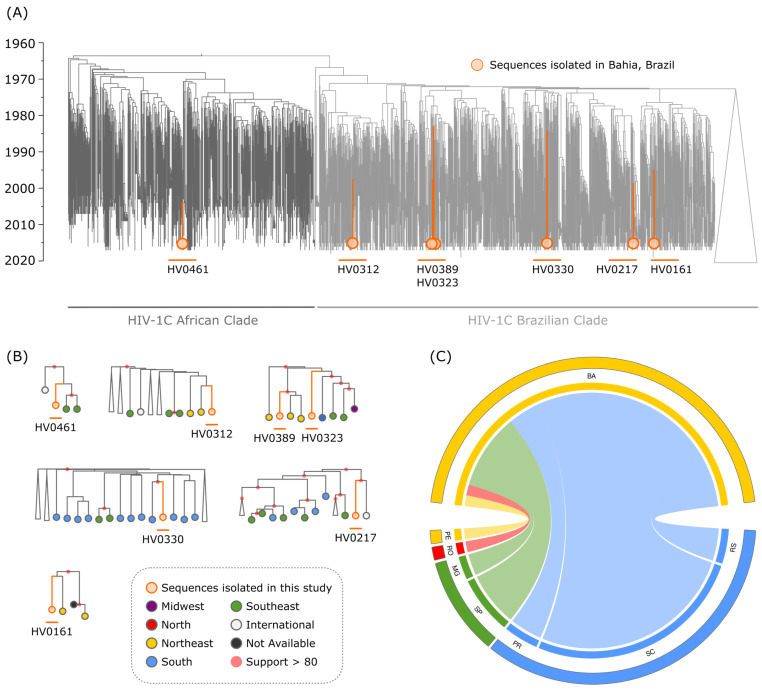
Phylogenetic reconstructions including seven HIV-1 subtype C sequences isolated in Salvador, Brazil. (**A**) Maximum likelihood phylogenetic reconstruction of 3631 HIV-1 subtype C sequences (partial pol). Orange branches denote the seven sequences isolated in Salvador (sample name is provided). Dark gray branches denote the African Clade, while light gray marks the Brazilian Clade. (**B**) Expanded clades are colored according to the Brazilian geographic region where the sequences were isolated. (**C**) Chord diagram representing the states of origin of the HIV-1 subtype C sequences identified in the state of Bahia. Figure includes the following Brazilian states colored according to the geographic region: RS (Rio Grande do Sul), SC (Santa Catarina), PR (Paraná), SP (São Paulo), MG (Minas Gerais), RO (Rondônia), PE (Pernambuco).

The phylogenetic reconstruction of HIV-1C reveals two major, distinct clades: the African Clade, primarily composed of sequences isolated from African countries, and the Brazilian Clade, a well-known monophyletic group encompassing all sequences isolated in Brazil (Figure 1) [17,18,19,20]. Previous evolutionary analyses have established that the Brazilian clade emerged as a monophyletic subcluster within the subtype C Eastern Africa Clade in the early 1980s [21,22]. The HIV-1C epidemic in Brazil began with the introduction of a single founder strain in the Southern region, which has since been sustained by ongoing transmission events.

Six of the seven HIV-1C sequences isolated in this study were found within the Brazilian Clade, but did not cluster together, suggesting the existence of multiple introductions of the virus in the state (Figure 1). To further explore the spread of subtype C in the state and its connections with the other part of Brazil, we used “mugration” analysis to infer the ancestral location of all HIV-1C sequences isolated in Bahia (n = 59). We found that the states in the Southern region, particularly Santa Catarina, accounted for more than half of the HIV-1C introductions in Bahia (Figure 1C). These results support the hypothesis of a discrete spread of subtype C into the Northeast region, originating from the South [23]. Santa Catarina is likely the primary source of transmission, while São Paulo may serve as a secondary hub, receiving cases from the Southern states and transmitting them to the Northeast [6].

Notably, one of the seven sequences isolated in this study was found within the African Clade (HV0461). This sequence, identified in Salvador, clustered in a well-supported clade (98.1 aLRT) that includes two other sequences from Brazil (both in São Paulo), suggesting possible sustained transmission of a HIV-1C lineage from Africa. Through a maximum likelihood approach, we estimated that this HIV-1C lineage emerged from the African continent around 1988. This cluster should be closely monitored in future studies to assess its potential impact on HIV transmission dynamics in Brazil and to understand the evolutionary trajectory of this lineage. Additionally, we identified 31 other Brazilian sequences intermingled within the African Clade, suggesting several independent introductions of the HIV-1C virus into Brazil. However, no evidence of sustained transmission originating from these introductions was observed.

In conclusion, our study provides critical insights into the phylogenetic dynamics of HIV-1C in Brazil, highlighting the expansion of HIV-1C to the Northeast region. The significant role of the Southern region, particularly Santa Catarina, in the spread of subtype C to the Northeast underscores the complexity of the HIV-1 epidemic in Brazil. Furthermore, the presence of Brazilian sequences within the African Clade suggests multiple independent introductions, emphasizing the need for ongoing surveillance and research to monitor these trends. Future studies should aim to elucidate the public health implications of these findings, as understanding the genetic diversity and transmission pathways of HIV-1C is crucial for effective control measures and targeted prevention efforts.

## Figures and Tables

**Table 1:** Clinical and demographic characteristics of seven HIV-1 patients infected with subtype C and diagnosed in Bahia, Brazil.

Patient ID	Gender	Age	Viral Load (Copies/mL)	CD4 Cell Count/m^3^	CD8 Cell Count/m^3^	CD4/CD8 Ratio	CD45 Cell Count/m^3^
HV0161	Female	39	41,015	87	804	0.11	1446
HV0217 *	Male	28	NI	NI	NI	NI	NI
HV0312	Male	27	30,400	455	1066	0.43	2770
HV0323	Female	30	407	370	741	0.5	1848
HV0330 *	Male	40	NI	NI	NI	NI	NI
HV0389	Male	26	15,811	433	1313	0.33	2679
HV0461	Female	44	256,632	43	813	0.05	1335

NI = not informed; * Patient did not return for follow-up after diagnostic.

## Data Availability

The new sequences have been deposited in NCBI GenBank under accession numbers MW596964, MW597005, MW597019, MW597021, MW597024, MW597044, and MW597061.
